# *De Novo* Transcriptome Assembly from Inflorescence of *Orchis italica*: Analysis of Coding and Non-Coding Transcripts

**DOI:** 10.1371/journal.pone.0102155

**Published:** 2014-07-15

**Authors:** Sofia De Paolo, Marco Salvemini, Luciano Gaudio, Serena Aceto

**Affiliations:** Department of Biology, University of Naples Federico II, Napoli, Italy; Niels Bohr Institute, Denmark

## Abstract

The floral transcriptome of *Orchis italica*, a wild orchid species, was obtained using Illumina RNA-seq technology and specific *de novo* assembly and analysis tools. More than 100 million raw reads were processed resulting in 132,565 assembled transcripts and 86,079 unigenes with an average length of 606 bp and N50 of 956 bp. Functional annotation assigned 38,984 of the unigenes to records present in the NCBI non-redundant protein database, 32,161 of them to Gene Ontology terms, 15,775 of them to Eukaryotic Orthologous Groups (KOG) and 7,143 of them to Kyoto Encyclopedia of Genes and Genomes (KEGG) pathways. The *in silico* expression analysis based on the Fragments Per Kilobase of transcript per Million mapped reads (FPKM) was confirmed by real-time RT-PCR experiments on 10 selected unigenes, which showed high and statistically significant positive correlation with the RNA-seq based expression data. The prediction of putative long non-coding RNAs was assessed using two different software packages, CPC and Portrait, resulting in 7,779 unannotated unigenes that matched the threshold values for both of the analyses. Among the predicted long non-coding RNAs, one is the homologue of *TAS3*, a long non-coding RNA precursor of *trans*-acting small interfering RNAs (ta-siRNAs). The differential expression pattern observed for the selected putative long non-coding RNAs suggests their possible functional role in different floral tissues.

## Introduction

The family Orchidaceae is one of the most widespread and species-rich plant families, including more than 25,000 species adapted to different habitats and displaying highly specialized morphological and physiological characteristics [1]. The evolutionary success of orchids has been attributed to different causes: epiphytism, highly diversified pollination strategies, natural selection, genetic drift and the unique features of their zygomorphic flowers [2], [3], [4]. Although extremely diversified, orchid flowers share a common architecture of the floral organs. They are organized into three sepals termed outer tepals and three petals distinguished in two inner lateral tepals and one inner median tepal (lip or labellum). The inner whorl, the column, is a fusion of male and female reproductive tissues, at the apex of which are located the pollen grains aggregated into pollinia [5]. The ovary is positioned at the base of the column, and its maturation is triggered by pollination [6].

**Figure 1:**
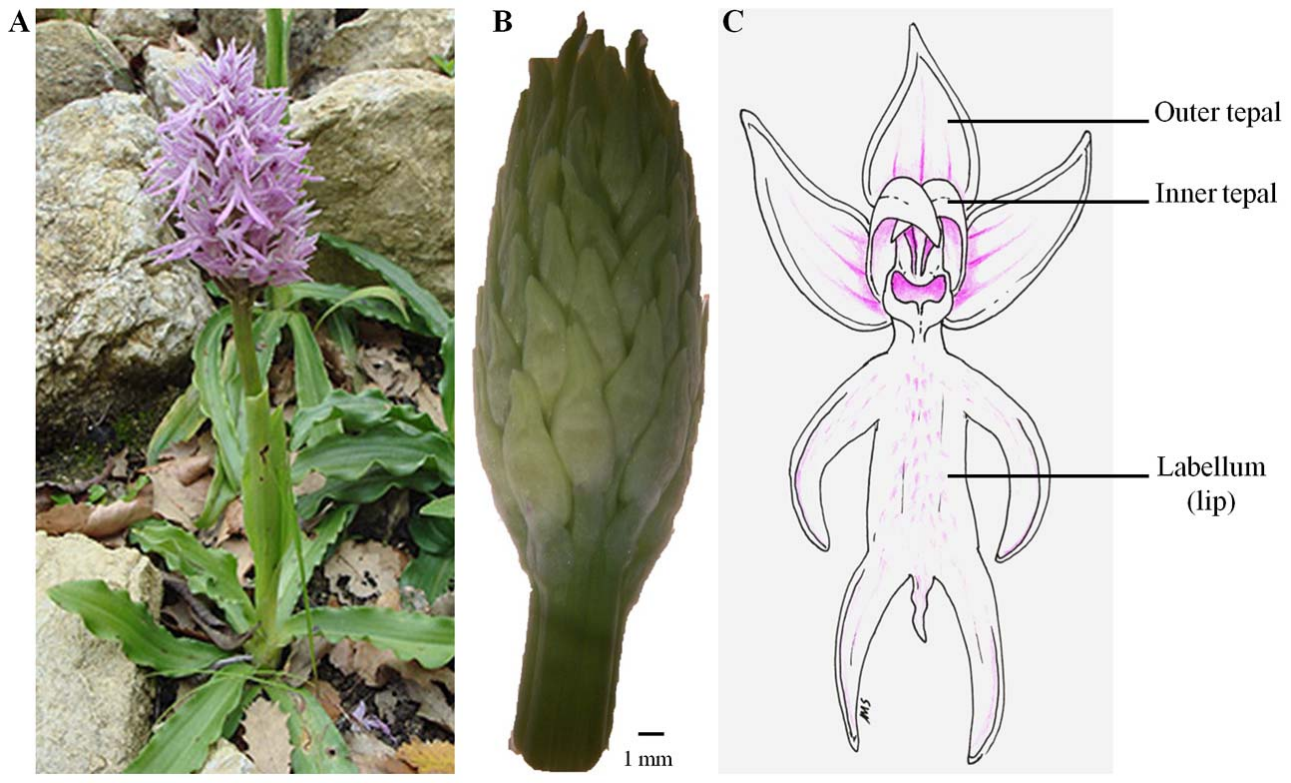
Inflorescence of *O. italica* after (A) and before (B) anthesis. (C) Schematic diagram of a single floret of *O. italica*.

*Orchis italica* belongs to the sub-family Orchidoideae (tribe Orchidinae). It is a diploid species (2n = 42) [7], with an Eurasian geographical distribution [8]. The inflorescence shape is short, dense and oval, with numerous pink flowers that start flowering from the base of the inflorescence and progress upwards (Figure 1 A) [9]. The study of flower development in *O. italica* is particularly challenging due to its difficulty to germinate *in vitro*. Nevertheless, the knowledge of the molecular mechanisms underlying flower development in *O. italica* has great relevance in comparative evolutionary studies with other orchid and non-orchid species.

In recent years, an increasing number of studies have been conducted on the genes and microRNAs (miRNAs) involved in the flower development of *O. italica* [4], [9], [10], [11], [12], [13], [14], [15], [16], [17] and other orchid species [18], [19], [20], [21], [22], [23], [24], [25], [26], [27], [28], [29], [30], [31]. Recently, next generation sequencing approaches have been applied to identify genes associated with flowering in some orchid species belonging to the Epidendroideae sub-family (genera *Phalaenopsis*, *Cymbidium*, *Oncidium*, *Ericina*) [29], [32], [33], [34], [35], [36], whereas similar studies in the Orchidoideae sub-family have been limited to *Ophrys* [37]. Although all of these RNA-seq studies include comprehensive analyses of coding and/or small non-coding RNAs in orchid species, none of them focus on the long non-coding RNAs (lncRNAs). In eukaryotes, transcriptome analyses have shown that approximately 90% of the DNA in genomes can be transcribed, even though only a very small percentage of the transcripts encode for protein products [38]. The non-coding RNAs are a large class of transcripts, with both housekeeping (e.g., ribosomal and transfer RNAs) and regulatory functions [38]. Based on their size, the regulatory non-coding RNAs are classified into small and long [39], [40]. The plant small RNAs (e.g., siRNAs and miRNAs) are involved in regulating gene expression through the cleavage or translational repression of a target transcript [41], [42]. However, the functions of lncRNAs are only recently becoming clearer. In some plant species, such as *Arabidopsis*, wheat and millet, it has been demonstrated that lncRNAs are involved in the response to stress [39], [40], [43] and in the silencing of the *FLOWERING LOCUS C* gene during vernalization [44], [45].

The involvement of plant lncRNAs in the control of flowering time led us to hypothesize that these non-coding RNAs may also be involved in other aspect of flowering, such as flower development.

The aim of this study is to expand the currently available sequence data for orchid species belonging to the Orchidoideae sub-family by producing a reference transcriptome of inflorescence tissue of *O. italica* and analyzing both the coding and long non-coding transcripts potentially involved in flower development.

## Materials and Methods

### Library construction and sequencing

Total RNA was extracted from 10 pooled florets collected from the bottom of a single unpollinated inflorescence of *O. italica* before anthesis (floral bud ∼9 mm diameter size, Figure 1 B) using the TRIzol Reagent (Ambion) and treated with DNase. The collected florets displayed approximately the same size and could be considered in the same developmental stage, with all floral organs formed (cell division is completed but cell distension is still occurring). The RNA was quantified using a NanoDrop 2000c spectrophotometer (ThermoScientific), and its integrity was assessed by measuring the RNA integrity number (RIN) using a 2100 Bioanalyzer (Agilent). The Illumina RNA-seq experiment was conducted at Genomix4Life S.r.l. (Salerno, Italy) following the Illumina TruSeq Stranded sample preparation protocol. Paired-end (PE) strand-specific sequencing was performed on an Illumina HiSeq 1500 instrument following the supplier-provided protocols and generating 100 nt long reads. The raw reads were deposited in the GenBank Short Read Archive under the accession SRX516901.

### Transcriptome *de novo* assembly

Quality control was performed by sliding window analysis and adapter trimming of the raw reads using Trimmomatic [46]. Contaminant reads matching rRNAs, tRNAs, *Cymbidium* mosaic virus (accession number NC_001812), *Odontoglossum* ringspot virus (NC_001728) or *E. coli* were removed using the Bowtie aligner v 1.0 [47] allowing for 2 mismatches (-v 2). The obtained high quality, cleaned reads were assembled using Trinity 2013.11.10 [48], [49] with the fixed default k-mer size of 25, minimum contig length of 200, maximum length expected between fragment pairs of 500 and a butterfly HeapSpace of 20 Gb. The similarity clustering of the assembled transcripts was performed using CD-HIT EST [50] with an identity cut-off of 85%.

### Functional annotation

The assembled transcripts were annotated using FastAnnotator [51] with the default search parameters. FastAnnotator performs a LAST search to find the best hits in the NCBI non-redundant protein database (nr), assigns the Gene Ontology terms (GO) using the Blast2Go software [52], and identifies the Pfam protein domains and the Enzyme Commission (EC) numbers.

The KOG (Eukaryotic Orthologous Groups) [53] annotations were identified by performing a RPSTBLASTN search [54] against the NCBI KOG database with a significance cut-off E-value of 1e^−5^.

The Kyoto Encyclopedia of Genes and Genomes (KEGG) pathways [55], [56] were inferred using the pathways of *Arabidopsis thaliana* and *Oryza sativa* as the reference (cut-off E-value 1e^−5^).

A BLASTX search (cut-off E-value 1e^−5^) was performed against the Transcription Factor (TF) databases of *A. thaliana* and *O. sativa* downloaded from PlantTFDB v3.0 [57].

*Arabidopsis* and *Oryza* were chosen as they represent dicot and monocot model species, respectively.

To evaluate the expression level of the assembled transcripts, the Fragments Per Kilobase of transcript per Million mapped reads (FPKM) were calculated using RSEM [58]. This software calculates the FPKM values for each assembled transcript by normalizing the counts of the PE reads to both the length of the transcript and the total number of mapped reads in the sample [59]. Transcripts with FPKM values above 100 were further investigated for the GO enrichment analysis. The number of GO terms annotated at level 2 between the reference transcriptome and the selected most expressed unigenes were compared by the Fisher exact test using R v 3.0.1 (http://www.r-project.org/).

Among the assembled protein coding transcripts, 10 were selected to compare their abundance estimated *in silico* (FPKM) with that measured by quantitative RT-PCR. Total RNA (1 µg) extracted from inflorescence of *O. italica* was reverse transcribed using the Advantage RT-PCR kit (Clontech) and an oligo dT primer. Specific primer pairs (Table 1) were used to amplify 30 ng of the first strand cDNA. The reactions were conducted in technical and biological triplicates (three biological samples and three technical triplicates for each) following the conditions previously reported [15]. The Real-Time PCR Miner online tool [60] was used to calculate the PCR efficiency (E) and optimal threshold cycle (C_T_) for each well. The mean relative expression ratio (Rn) and standard deviation of the selected coding transcripts was calculated using the *actin OitaAct* gene [GenBank: AB630020] as the endogenous control applying the formula Rn = R0_target_/R0_control_ = (1+E_target_)^−CTtarget^/(1+E_control_)^−CT control^.

**Table 1:** Protein coding unigenes selected for the expression analysis validation.

Encoded gene	ABCDE class	GenBank ID	Unigene name	Primer (5′-3′)	FPKM	FPKMn	Rn
***OitaDEF4***	B (*DEF*-like clade 4)	AB857729	comp900_c0_seq1	TCTGAGGAGGGATGTAAGACAGAGGA	181.29	64.29	243.76
				ATAGGTGTCTGTCTGCGTACTGATTA			
***OrcPI2***	B (*GLO*-like)	AB537504	comp1173_c0_seq1	GAGAGTACGCACCGCCACCG	134.3	47.62	239.04
				GCTGGATGGGCTGCACACGA			
***OitaDEF3***	B (*DEF*-like clade 3)	AB857728	comp7668_c0_seq1	CCTGAGGAGGGAGATAAGGCAGAGAA	112.74	39.98	58.50
				GTATGTATCAGTCTGGGTGCTAATGC			
***OrcPI***	B (*GLO*-like)	AB094985	comp1989_c0_seq1	CCCAGAATATGCGGACCAGATGCC	108.63	38.52	126.00
				TGGGCTGGAAAGGCTGCACG			
***OitaDEF1***	B (*DEF*-like clade 1)	AB857726	comp3831_c0_seq1	CCTTCGCAGGGAGATAAGGCAAAGGA	56.84	20.16	82.81
				GTAAGTGTCTGTTTGCGTGGCGATCA			
***OitaAG***	C (*AG*-like)	JX205496	comp7958_c0_seq1	TCTGCAACAAATGCGCAGTAT	40.55	14.38	23.23
				AAGCTTGTGATTTGCTGTCGAA			
***OitaSTK***	D (*STK*-like)	JX205497	comp3859_c0_seq1	CGGAGCTACACGATGAAAGTATGT	37.75	13.39	36.35
				CCGCGCCCTCTCGTTTT			
***OitaAP2***	A (*AP2*-like)	KF152921	comp8045_c0_seq1	TGTGTACCCCGGATTATTTCCT	26.78	9.50	9.60
				TTTCTGGGGCCAAGTGGTCATGGT			
***OitaDEF2***	B (*DEF*-like clade 2)	AB857727	comp22604_c0_seq1	CCTTCGGAAGGAGATAAGGCAGAGGA	5.2	1.84	44.06
				GTAAGTGTCAGTTTGGGTAGCGATCA			
***OitaAct***		AB630020	comp44267_c0_seq1	TCGCGACCTCACCAATGTAC	2.82	1.00	1.00
				CCGCTGTAGTTGTGAATGAATAGC			

The name of the gene family and clade are reported in parenthesis. The sequences of the primer pairs used in the real-time PCR experiments are indicated, as well as the FPKM counts for each assembled unigene and their respective normalized value (FPKMn) relative to the *actin* counts. Rn indicates the relative expression value obtained in the real-time PCR experiments.

### Analysis of the non-coding transcripts

The analysis of the potential non-coding transcripts was performed using two prediction software packages, Coding Potential Calculator (CPC) [61] and Portrait [62]. CPC assesses the coding potential of a transcript by examining the extent and quality of the ORF in a transcript and then performing a BLASTX search against the UniProt Reference Clusters. A positive value of the coding potential score in CPC indicates that a specific transcript potentially encodes for a protein, whereas a negative value predicts a potential non-coding transcript. Portrait is particularly suitable for the analysis of non-model organisms. The transcripts and their predicted putative proteins are evaluated by a support vector machine and no homology information is required. In Portrait, the non-coding potential of a transcript is expressed as a percentage. To extract potential non-coding transcripts from the assembled transcriptome, we applied the arbitrary threshold values ≤−0.8 for the CPC coding potential score and ≥95% for the Portrait non-coding probability.

Ten unannotated transcripts were selected for validation experiments. Specific primer pairs (Table 2) were designed and used to amplify the cDNA obtained from the total RNA of inflorescence of *O. italica*. The specific primer pairs were used to amplify 30 ng of first strand cDNA using the LongAmp Taq PCR Kit (New England Biolabs). The amplification products were cloned into the pGEM-T Easy vector (Promega) and sequenced using the plasmid primers T7 and SP6. The sequencing reactions were run on an ABI 310 Automated Sequencer (Applied Biosystems). The obtained nucleotide sequences were aligned to their respective transcripts of the transcriptome of *O. italica*.

**Table 2:** Putative long non-coding unigenes selected for the expression analysis and nucleotide sequence of the primer pairs used in the amplification experiments.

Unigene name	Length	Primer (5′-3′)	Amplicon length	FPKM	CPC	Portrait
**comp48038_c0_seq1**	300	ACACCTTAATACAACCCTAAACCCT	224	2.67	−1.62	96.26
		TAACACCGGGGCAATGTCTT				
**comp1308_c0_seq1**	1246	ATCTGCAACGGGGGCATAAA	917	435.18	−1.03	32.33
		TGTTTCGCGGTCAGATCCAA				
**comp0_c0_seq1**	597	AAGCCTGCTGCCTTCGTTAT	386	20357.49	−0.31	4.87
		CAACACAGACTGGCTGGCTA				
**comp3328_c0_seq1**	214	CGTTCTGGTGGAGTTTGTCC	173	87.04	−1.13	95.64
		AATTGGCATGCATCAAGAAA				
**comp1231_c0_seq1**	772	AACGAATCCTGACCGCAGTT	308	61.91	−1.03	96.08
		ACTCATTTGCGGTCCTCCTG				
**comp3311_c0_seq1**	894	CCTCGGCCTAAAGAGGTAGC	360	52.42	−1.10	96.22
		ACAGTTGACCATCGCTCTCC				
**comp6669_c0_seq1**	217	ACACAGCAGCAAGTTGGTCTT	126	51.02	−1.32	95.00
		TGACCCCCAACACACAACAG				
**comp4129_c0_seq1**	611	CAGACATGGCAGAACGAAGA	202	46.77	−1.19	96.38
		AGCCGGAAGATAAGCTGACA				
**comp15481_c0_seq1**	2888	GAAGAAGCAATGAGCCCCCT	924	9.90	−1.33	84.89
		CAACCTACCAGTTCCGGTCC				
**comp134696_c0_seq1**	203	GGCGTTATCCTGATTGAGCTTTTC	203	0.64	−0.92	96.89
		CAGCTCAGGAGGGATAGAAGGGGG				

The CPC and Portrait columns indicate the coding potential score and the non-coding probability as a percentage, respectively.

The expression pattern of the selected putative long non-coding transcripts was verified in different tissues of *O. italica* (outer and inner tepals, lip, column, unpollinated ovary, leaf) by quantitative RT-PCR experiments as described above. Differences in the relative expression levels of the selected non-coding transcripts in the various tissues were assessed by ANOVA followed by the Tukey HSD post hoc test. The real-time PCR product from one sample for each non-coding transcript was cloned and sequenced to exclude the presence of amplification artifacts.

## Results and Discussion

### Illumina sequencing and *de novo* assembly

The inflorescence transcriptome of *O. italica* was generated starting from high quality total RNA (RIN = 9.0) extracted from inflorescence before anthesis. The cDNA library was sequenced, resulting in ∼94 million PE 100-bp reads of good quality (Phred quality score ≥33) and without contaminants (Table 3), which represented 86.2% of the original reads.

**Table 3:** Sequence assembly summary statistics.

	Number	N_50_	Mean	Min	Max	>1000	>2000	>3000	>5000	>10000
**Assembled transcripts** 1	132,565	786	564	201	12,047	18,004	4,357	1,210	143	3
**Unigenes**	86,079	956	606	201	12,047	13,996	3,928	1,185	140	3

Mean, Min and Max indicates the average, minimum and maximum length expressed in base pairs.

1 The starting reads were 108,911,910. After contaminant cleaning, 108,738,395 reads were obtained. Quality checking and adaptor trimming resulted in 93,926,808 reads that were used to assemble the transcriptome.

**Figure 2:**
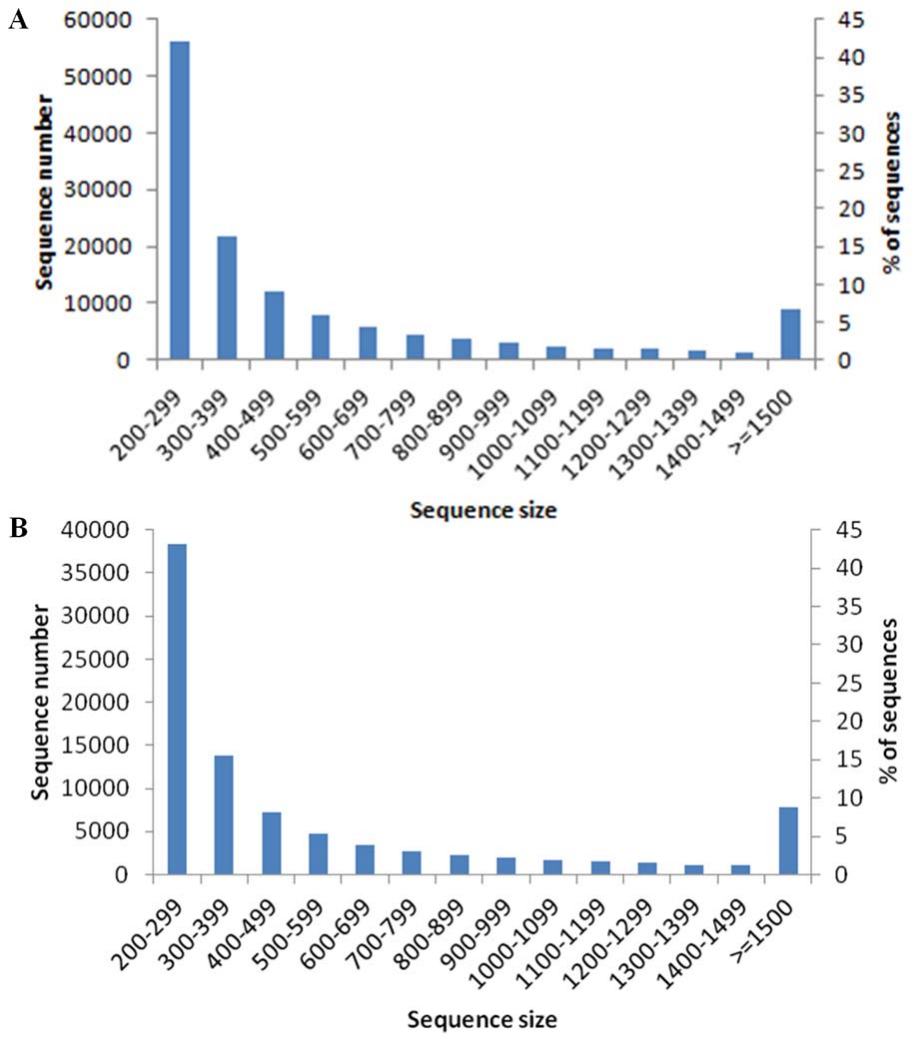
Size distributions of the assembled transcripts (A) and unigenes (B) of the inflorescence of *O. italica*. The length ranges are indicated in base pairs.

As the assembled genome of *O. italica* is not available, the cleaned reads were processed using a *de novo* approach. The *de novo* assembler Trinity [49] produced 132,565 assembled transcripts that were clustered into 86,079 not redundant transcripts (unigenes) based on their sequence identity (set to 85%) (File S1). The mean size of the unigenes was 606 bp, spanning from 201 to 12,047 bp, and the N50 value was 956 (Table 3). Figure 2 shows the size distribution of the assembled transcripts (A) and unigenes (B). Although the most abundant class of both the transcripts and unigenes fell in the size range between 200 and 300 bp (42.2% of the transcripts and 43.1% of the unigenes), 18,031 transcripts were more than 1,000 bp in length (13.6%), and 42,876 were more than 500 bp (32.3%). Among the unigenes, 14,012 were more than 1,000 bp (16.3%), and 28,589 (33.2%) were more than 500 bp.

The number of transcripts and unigenes assembled for the inflorescence of *O. italica* is higher than those assembled with the same deep sequencing approach for the inflorescence of the orchid *Cymbidium ensifolium* (101,423 transcripts and 51,696 unigenes) [34] and similar or slightly lower than those assembled for mixed vegetative and reproductive tissues of *Cymbidium sinense* [33] and *Erycina pusilla* [36]. The other orchid transcriptomes currently available (*Oncidium* ‘Gower Ramsey’, *Phalaenopsis aphrodite* and *Ophrys*) were obtained by applying combined approaches of different next generation sequencing (NGS) techniques [32], [35], [37], resulting in transcriptomes composed of both contigs and singletons. For example, the inflorescence transcriptome of *Ophrys*, a mixture of *O. exaltata*, *O. garganica*, and *O. sphegodes*, includes 51,795 contigs (Illumina data) and 70,122 singletons (454 and Sanger data) [37]. In addition to the different NGS approaches used, the variation in the number of transcripts assembled in orchids could also be related to their great diversity in genome size. Currently, Orchidaceae are the angiosperm family with the most variable genome size, with 1C ranging from 0.33 to 55.4 pg [63]. For example, the mean genome size estimated for the genus *Orchis* is ∼8.6 Gb and for *Ophrys* is ∼10 Gb, whereas the genera of the subfamily Epidendroideae have smaller mean values (e.g., *Erycina* ∼1.7 Gb, *Oncidium* ∼3.3 Gb, *Phalaenopsis* ∼3.7 Gb and *Cymbidium* ∼4 Gb) [63]. Almost all these values are considerably higher than those of plant species with completely sequenced genomes like *Arabidopsis thaliana* (∼0.135 Gb) (http://www.arabidopsis.org/portals/genAnnotation/gene_structural_annotation/agicomplete.jsp), *Oryza sativa* (∼0.466 Gb) (http://btn.genomics.org.cn/rice) and *Zea mays* (∼2.4 Gb) (http://plants.ensembl.org/Zea_mays/Info/Index).

### Functional annotation

**Figure 3:**
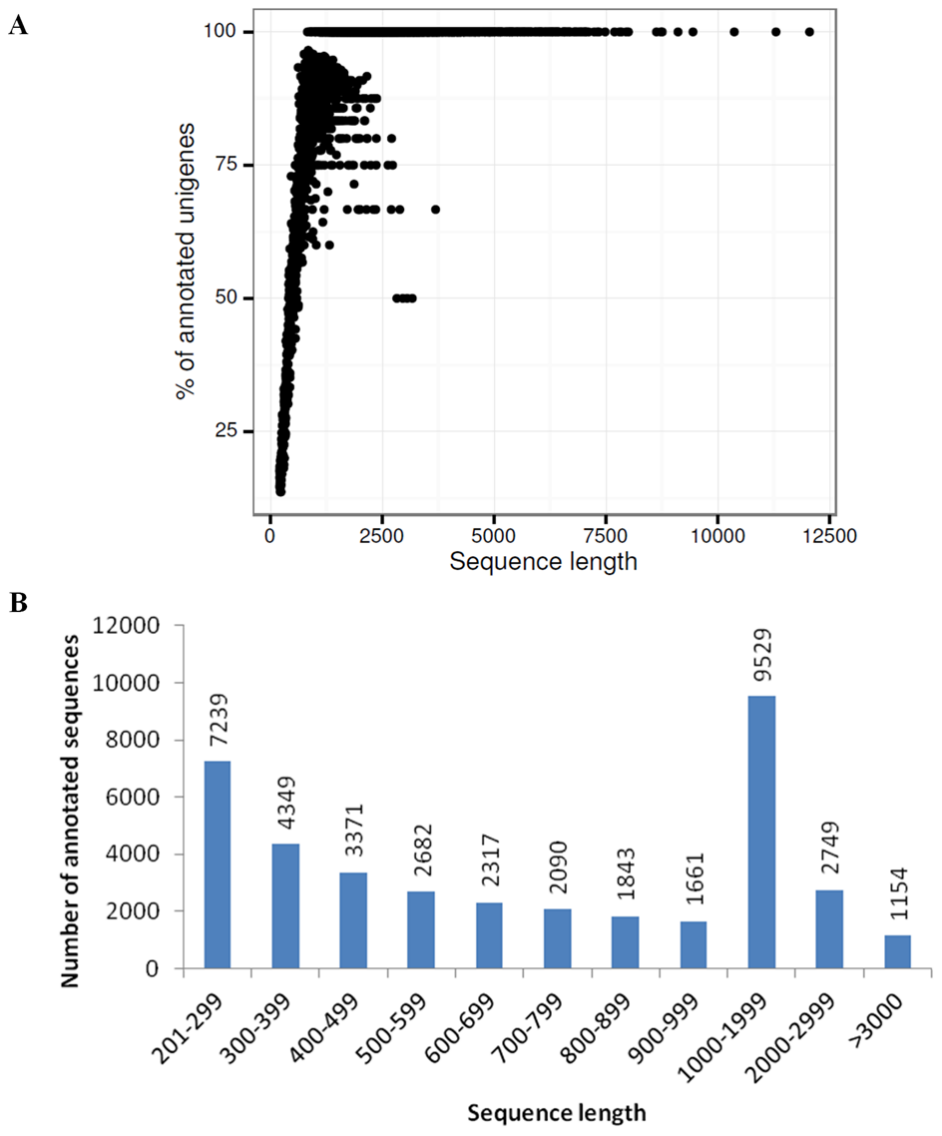
Size distribution of the annotated transcripts. (**A**) Relationship between the sequence length of the assembled unigenes and the percentage of annotations in the NCBI nr protein database. (**B**) Number of annotated unigenes for each size class. The lengths are indicated in base pairs.

The unigenes of the transcriptome of *O. italica* were annotated using the web platform FastAnnotator [51] (Table S1). Among all the unigenes, 38,984 (45.3%) matched at least one significant hit against the NCBI nr protein database (Table 4). This value is slightly lower than the number of annotated transcripts of *Ophrys* (44,034), *C. ensifolium* (41,873), *C. sinense* (41,687) and *E. pusilla* (39,839) and higher than those of *Oncidium* (22,810) and *Phalaenopsis* (22,234). The percentage of annotated unigenes of *O. italica* was positively correlated with the sequence length (Pearson correlation coefficient *r* = 0.57, p<0.001) (Figure 3 A). BLASTN analysis between the unannotated unigenes of *O. italica* and *Ophrys* resulted in 614 best reciprocal hits (1.3% of the unannotated unigenes of *O. italica*). These results indicate a high probability that most of the unannotated unigenes of *O. italica* are novel transcripts. Among the annotated sequences, the most abundant class (24.4%) had a sequence length between 1,000 and 2,000 bp (Figure 3 B).

**Table 4:** Statistics of the annotation results for the *O. italica* unigenes.

	All	NCBI-nr	GO	Enzyme (EC)	Pfam	KOG	KEGG
**Number of unigenes**	86,079	38,984	32,161	3,085	32,011	15,775	7,143
**% of unigenes**	100	45.3	37.4	3.6	37.2	18.3	8.3

Because *Ophrys* is the closest species to *Orchis* with a currently available transcriptome, the subsequent comparative analyses were based on to the transcriptome of inflorescence of *Ophrys*, even though the *Ophrys* transcriptome was obtained with a different sequencing approach than that applied in the present study.

**Figure 4:**
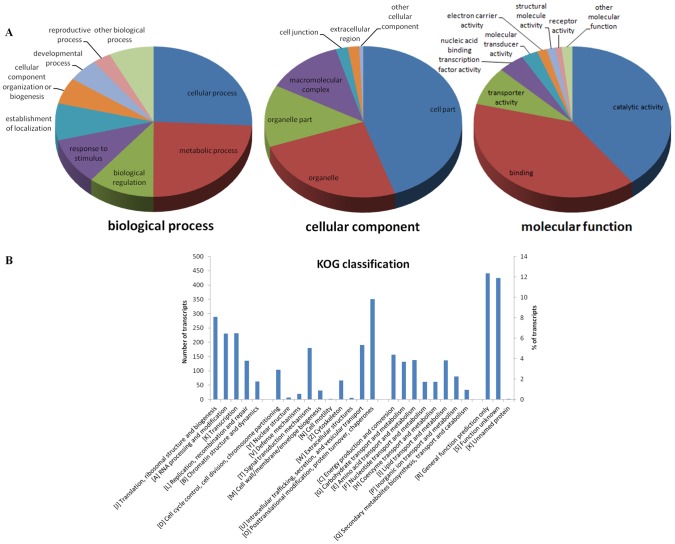
Functional annotations of the unigenes of *O. italica*. (**A**) Level 2 GO term distribution for the biological process, cellular component and molecular function categories. (**B**) KOG annotation.

FastAnnotator assigned the unigenes to possible functional categories, classifying them into three main classes of GO terms (Table S2). The most abundant class of functional annotation was biological process (28,558 unigenes), followed by molecular function (27,378) and cellular component (24,304). These numbers are higher than those reported for the transcriptome of *Ophrys*, where the most abundant class is molecular function (21,138 transcripts), followed by biological process (19,960) and cellular component (19,272). Figure 4 A shows the level 2 GO classification of the transcriptome of *O. italica*. Among the biological process terms, most of the unigenes were assigned to cellular and metabolic process (25.8% and 24.2%, respectively). In the molecular function category, the most abundant classes were binding (39.1%) and catalytic activity (39.7%), whereas cell part (44.8%) and organelle (24.8%) were the classes with the highest number of assigned unigenes in the cellular function category. The level 2 GO classification of the transcriptome of *O. italica* agrees with that reported for *Ophrys* [37].

An additional functional annotation of the unigenes of *O. italica* was performed searching for putative orthologs and paralogs within the KOG database [53]. A total of 15,775 unigenes (18.3%) were assigned to 26 eukaryotic orthologous groups (Table 4, Figure 4 B). The general (R, 12.3%) and unknown (S, 11.9%) functions were the most represented, followed by post-translational modifications, protein turnover and chaperones (O, 9.8%). The percentage of unigenes assigned to KOG terms and the relative abundance of each KOG group are in general agreement with those reported for *Ophrys* [37]. The only exceptions are the group S (function unknown), which is higher in *Orchis* than in *Ophrys* (∼6%), Q (secondary metabolites biosynthesis, transport and catabolism), which in *Orchis* is approximately 1/5 that in *Ophrys*, and T (signal transduction mechanisms), which in *Orchis* is approximately 1/2 that in *Ophrys*.

**Figure 5:**
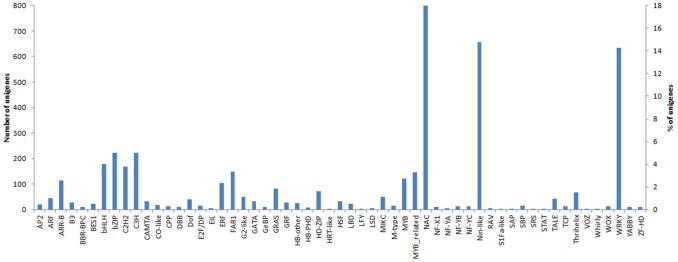
Transcription factor annotations of the unigenes of *O. italica* obtained from the plant TFDB.

To specifically identify transcription factors within the assembled transcripts of *O. italica*, the unigenes were used to perform a search against the Plant Transcription Factor Database, using *A. thaliana* and *O. sativa* as reference dicot and monocot species, respectively. A total of 4,095 unigenes (4.8%) matched with 57 plant transcription factor families (Figure 5), a number and percentage that is slightly higher than those reported for *Ophrys* (3,319 transcripts, 2.7% of the reference transcriptome), where 56 families were identified (the HRT-like family was not identified in *Ophrys*). In *O. italica*, the most abundant transcription factor families were NAC (18.1%), Nin-like (14.7%) and WRKY (14.3%), which is in partial agreement with the results obtained in *Ophrys*, where the transcription factor families most highly represented were WRKY (21.4%), NAC (7.8%) and NF-YA (12.5%); the Nin-like family was at 5.8% [37]. Other representative transcription factor families in *O. italica* were C3H, bZIP, bHLH, MYB and those involved in flower development (AP2, LFY, MIKC, TCP).

Among the assembled transcripts of *O. italica*, 32,011 (37.2%) matched with 7,208 protein domains in the Pfam database (http://pfam.sanger.ac.uk/) with coverage greater than 50% (Table 4). The PPR domain was the most highly represented, followed by RVT_2 and Pkinase (Table 5, Table S3). The PPR domain proteins are one of the largest plant protein families involved in different aspects of plant physiology and development. Their main function is to target different organellar transcripts, modulating their expression through RNA editing and/or regulation of the mRNA turnover and translation [64]. The RVT proteins are reverse transcriptases indicative of the presence of mobile elements such as retrotransposons or retroviruses. Their abundance in the transcriptome of *Orchis italica*, together with other abundant protein classes such as rve, gag_pre-integrs, and Retrotrans_gag, is related to the high number of mobile elements reported in the orchid genomes [14], [15], [65]. The protein kinases (Pkinase) are involved in cell proliferation, differentiation and death [66]. These protein classes are also highly represented in the transcriptome of *Ophrys*, as are other protein families such as the single-strand RNA binding proteins RRM, the WD domain G-beta repeats WD40, the cytochrome P450 and those related to the presence of mobile elements [37].

**Table 5:** Summary statistics of the Pfam domain annotations with occurrence >100.

Short name	Accession	Description	Occurrence
**PPR_2**	PF13041	Pentatricopeptide repeat family	825
**RVT_2**	PF07727	Reverse transcriptase (RNA-dependent DNA polymerase)	670
**Pkinase**	PF00069	Protein kinase domain	527
**rve**	PF00665	Integrase core domain	372
**ABC_tran**	PF00005	ATP-binding domain of ABC transporters	252
**MFS_1**	PF07690	Major facilitator superfamily	249
**LysR_substrate**	PF03466	LysR substrate binding domain	197
**Pkinase_Tyr**	PF07714	Tyrosine kinase	191
**RVT_1**	PF00078	Reverse transcriptase	183
**UBN2_3**	PF14244	gag-polypeptide of LTR copia-type	173
**AMP-binding**	PF00501	AMP-binding enzyme	167
**RRM_1**	PF00076	RNA recognition motif	167
**gag_pre-integrs**	PF13976	gag-pre-integrase domain	156
**WD40**	PF00400	WD40 repeat	144
**Tymo_45kd_70kd**	PF03251	Tymovirus 45/70Kd protein	140
**Retrotrans_gag**	PF03732	Retrotransposon gag protein	138
**BPD_transp_1**	PF00528	Binding-protein-dependent transport system inner membrane	131
**LRR_8**	PF13855	Leucine-rich repeat	127
**ACR_tran**	PF00873	AcrB/AcrD/AcrF family integral membrane proteins	124
**adh_short**	PF00106	Short-chain dehydrogenase	124
**Response_reg**	PF00072	Response regulator receiver domain	122
**Aldedh**	PF00171	Aldehyde dehydrogenase family	121
**DYW_deaminase**	PF14432	DYW family of nucleic acid deaminases	120
**Myb_DNA-binding**	PF00249	Myb-like DNA-binding domain	118
**zf-RING_2**	PF13639	RING finger domain	118
**p450**	PF00067	Cytochrome P450	116
**Abhydrolase_6**	PF12697	Alpha/beta hydrolase fold	114
**TonB_dep_Rec**	PF00593	TonB-dependent receptors	102
**Other domains**			26,022

In the transcriptome of *O. italica*, 3,085 transcripts (3.5%) had at least one enzyme hit in the Enzyme database (http://enzyme.expasy.org/) (Table 4). The unigenes of *O. italica* were also used to search for an alternative functional annotation in the KEGG database according to their involvement in biochemical pathways, resulting in 7,143 transcripts (8.3%) matching with 2,651 enzymes involved in essential biochemical pathways (Table 4 and 6). The highest number of matching transcripts and enzymes was involved in metabolism (carbohydrate, amino acid, lipid, energy, etc.), followed by genetic information processing, cellular processes, environmental information processing and organismal systems. The number of transcripts of *O. italica* involved in KEGG pathways and their assignment to specific sub-pathways are similar to those reported for *Ophrys* [37].

**Table 6:** Summary of the KEGG pathways analysis indicating the number (N) of unigenes and the number of corresponding enzyme matches.

KEGG pathway	N unigenes	N enzymes
**Metabolism**		
Global and overview maps	3032	1116
Carbohydrate metabolism	977	271
Amino acid metabolism	654	458
Lipid metabolism	433	165
Energy metabolism	278	61
Biosynthesis of other secondary metabolites	241	53
Metabolism of other amino acids	227	56
Metabolism of cofactors and vitamins	206	120
Nucleotide metabolism	192	73
Metabolism of terpenoids and polyketides	139	78
Glycan biosynthesis and metabolism	79	43
**Genetic Information Processing**		
Folding, sorting and degradation	178	24
Translation	109	37
Replication and repair	81	25
Transcription	48	8
**Cellular Processes**		
Transport and catabolism	112	28
**Environmental Information Processing**		
Signal transduction	87	25
**Organismal Systems**		
Environmental adaptation	58	10

The wide diversity of genes, GO terms, transcription factors, enzymes and biochemical pathways found indicates good coverage of the transcriptome of inflorescence of *O. italica*.

### Expression level analysis

The RSEM software [58] was used to evaluate the expression level of the unigenes of *O. italica* measured as FPKM. The obtained values of FPKM ranged from 0 to more than 20,000 (Table S4). The unigenes with FPKM values lower than 1 (36.4%) were considered as unexpressed, those with FPKM values between 1 and 10 (50.1%) were considered poorly expressed, those between 10 and 100 (12.2%) were considered moderately expressed, and those with FPKM values higher than 100 (1.3%) were considered highly expressed.

To validate the *in silico* expression analysis, nine unigenes annotated as genes involved in flower development and one housekeeping gene (Table 1) were selected, and their expression level was measured by real-time RT-PCR. All the selected unigenes encode transcriptional factors involved in the ABCDE model of flower development and all but one (*OitaAP2*) are MADS-box genes. The ABCDE model describes the integrated role of floral homeotic genes belonging to different functional classes in the specification of the flower organ identity [67], [68]. In brief, the identity of sepals is specified by A- and E-class genes; the formation of petals is determined by A-, B- and E-class genes; the identity of stamens is specified by B-, C- and E-class genes, that of carpels by C- and E-class genes. The development of ovules is regulated by D- and E-class genes. Although the ABCDE model is well conserved in a wide number of species, it does not fully fit in some species such as orchids and other non-grass monocots. For example, analyses of B-class genes in orchids revealed the expansion of their expression pattern to the first floral whorl (the outermost). This feature may explain the presence of petaloid sepals (tepals) in orchids [69].

**Figure 6:**
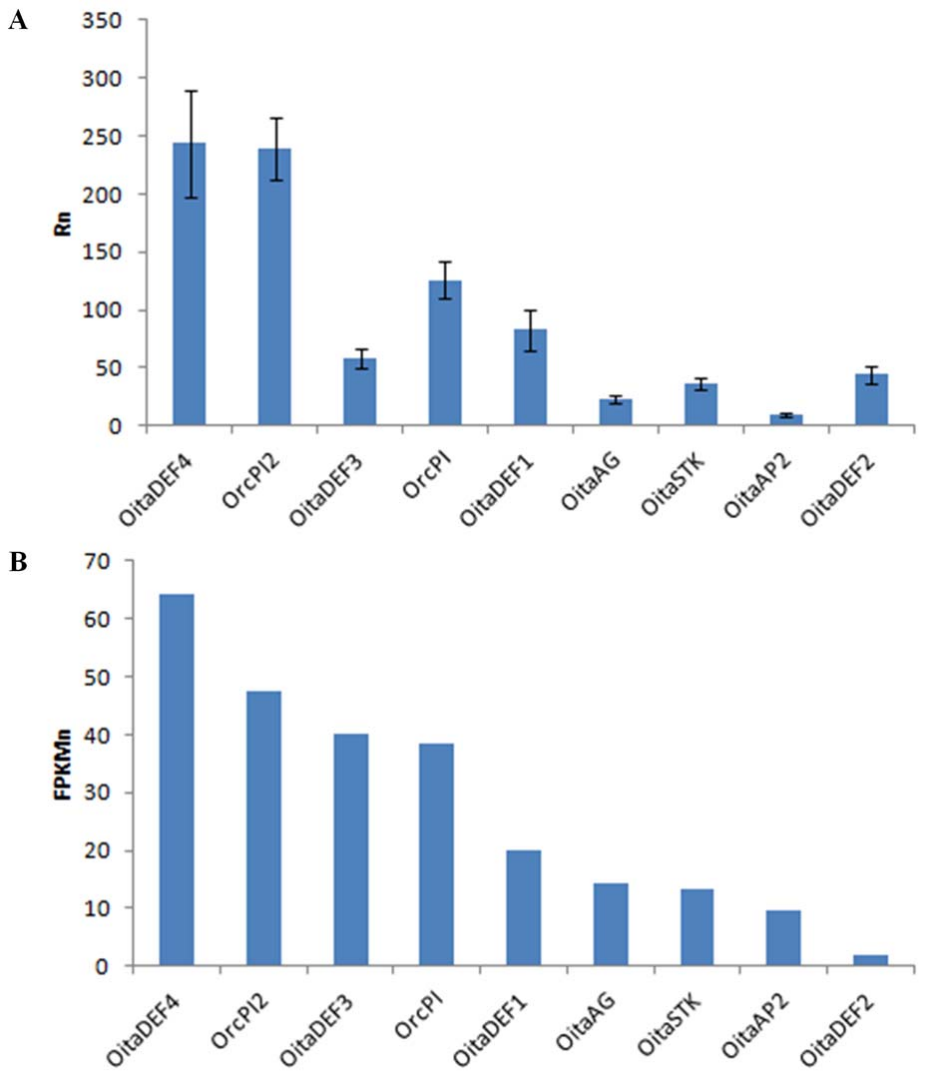
Relative expression levels of selected protein coding unigenes of *O. italica* assessed by real-time PCR analysis of inflorescence tissue (A) and by normalized FPKM counts (B). Both measures were normalized relative to the actin levels. The bars indicate the standard deviation.

The mean Rn values of the selected genes were obtained by dividing the R0 values of each gene by the R0 value of the reference gene (*actin*). They were compared to the normalized FPKM values (FPKMn), which were obtained by dividing the FPKM value of each unigene by the FPKM value of *actin*. The Pearson correlation coefficient showed a strong positive correlation between the two datasets (*r* = 0.87, p = 0.002) (Figure 6, Table 1), demonstrating that the FPKM values of the *de novo* assembled transcriptome of *O. italica* represent a good approximation of the real expression level of the transcripts in the inflorescence tissue.

**Figure 7:**
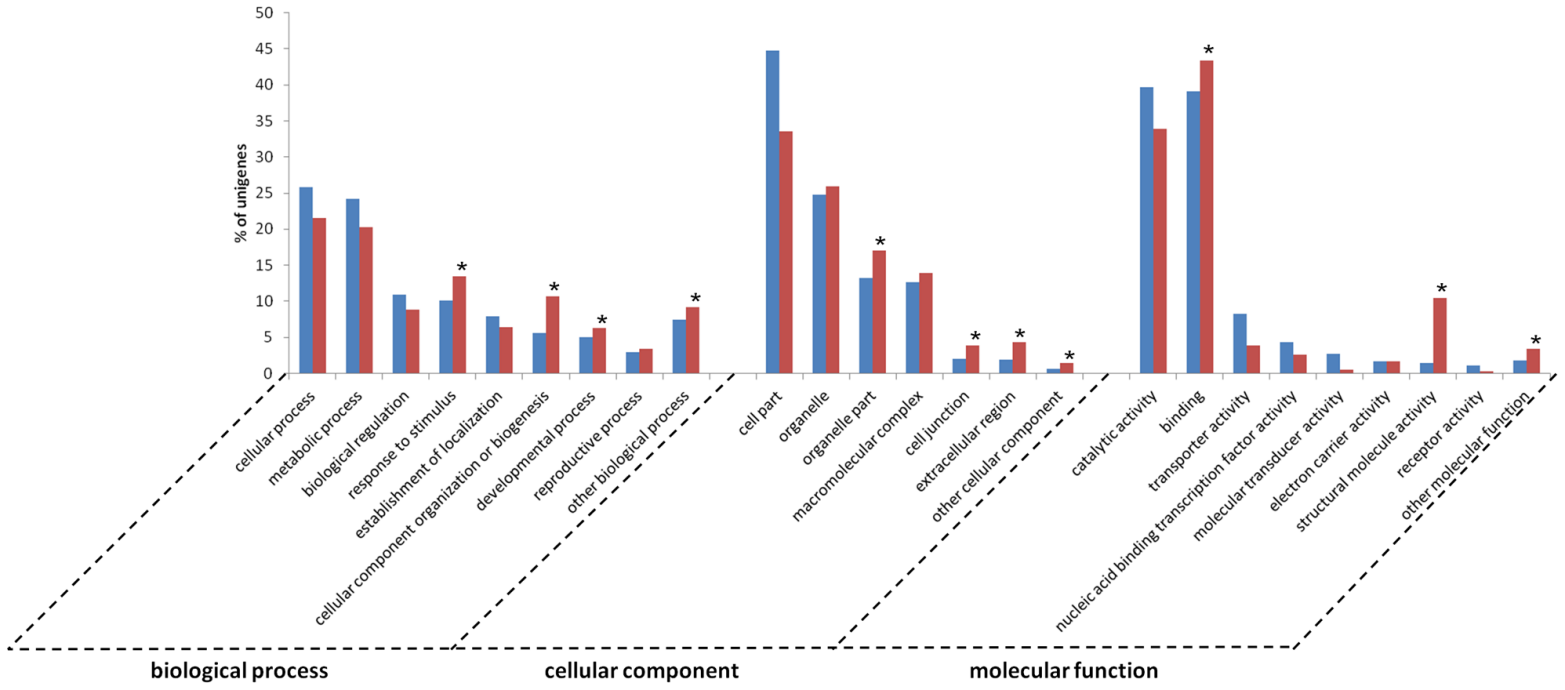
Comparison of the level 2 GO annotations between the reference transcriptome of *O. italica* (blue) and the 1,144 unigenes with FPKM counts greater than 100 (red). Asterisks indicate the significantly enriched GO terms among the most expressed unigenes (Fisher exact test *p*<0.05).

A GO enrichment analysis was performed to determine if the 1,144 most expressed unigenes of *O. italica* (FPKM >100) were enriched for in any GO term (Figure 7, Table S5). Among the biological processes, a significant enrichment was observed for response to stimulus (GO:0050896), cellular component organization or biogenesis (GO:0071840) and developmental process (GO:0032502). The organelle part (GO:0044422), cell junction (GO:0030054) and extracellular region (GO:0005576) were the cellular component categories significantly more represented in the most expressed transcripts than in the whole transcriptome. Finally, among the molecular functions, binding (GO:0005488) and structural molecule activity (GO:0005198) showed a statistically supported enrichment.

### Non-coding transcripts

In recent years, increasing interest has been focused on the study of the plant long non-coding RNAs (lncRNAs) and their involvement in regulatory processes such as development, response to stimuli and stress tolerance [39], [40], [43], [44]. The NGS approach and the development of *ad hoc in silico* analysis tools has greatly enhanced the ability to predict potential lncRNAs that can be functionally characterized *in vivo*. Currently, lncRNAs in orchids are completely unknown.

The absence of the assembled genome of *O. italica* (or of other orchid species) makes it difficult to approach the study of the lncRNAs in this species because it is impossible to determine whether the putative long non-coding sequences are 5′/3′UTRs of transcripts not fully assembled or true lncRNAs. However, a preliminary analysis was conducted to identify the putative lncRNAs assembled in the inflorescence transcriptome of *O. italica*. The 47,097 unannotated unigenes were analyzed to predict their coding potential using two different software packages: Coding Potential Calculator, or CPC [61], which uses machine-learning methods and comparative genomics, and Portrait [62], which uses a support vector machine and is optimized for non-model organisms. The arbitrary threshold values for the significance of the prediction were set to ≥95% (the Portrait non-coding probability) and ≤−0.8 (the CPC coding potential score). The prediction resulted in 45,266 (CPC) and 7,888 (Portrait) potential non-coding transcripts, with 7,779 transcripts matching both thresholds (Table S6).

Among the transcripts lacking a functional annotation, 10 were selected to verify whether they were true transcripts and to exclude them if they were assembly artifacts (Table 2). Seven of the selected transcripts matched both the CPC and Portrait threshold values, two matched only the CPC threshold and were chosen because their size exceeded 1,000 bp, and one did not match any threshold and was chosen because it showed the highest FPKM value (20,357) among the assembled transcripts.

**Figure 8:**
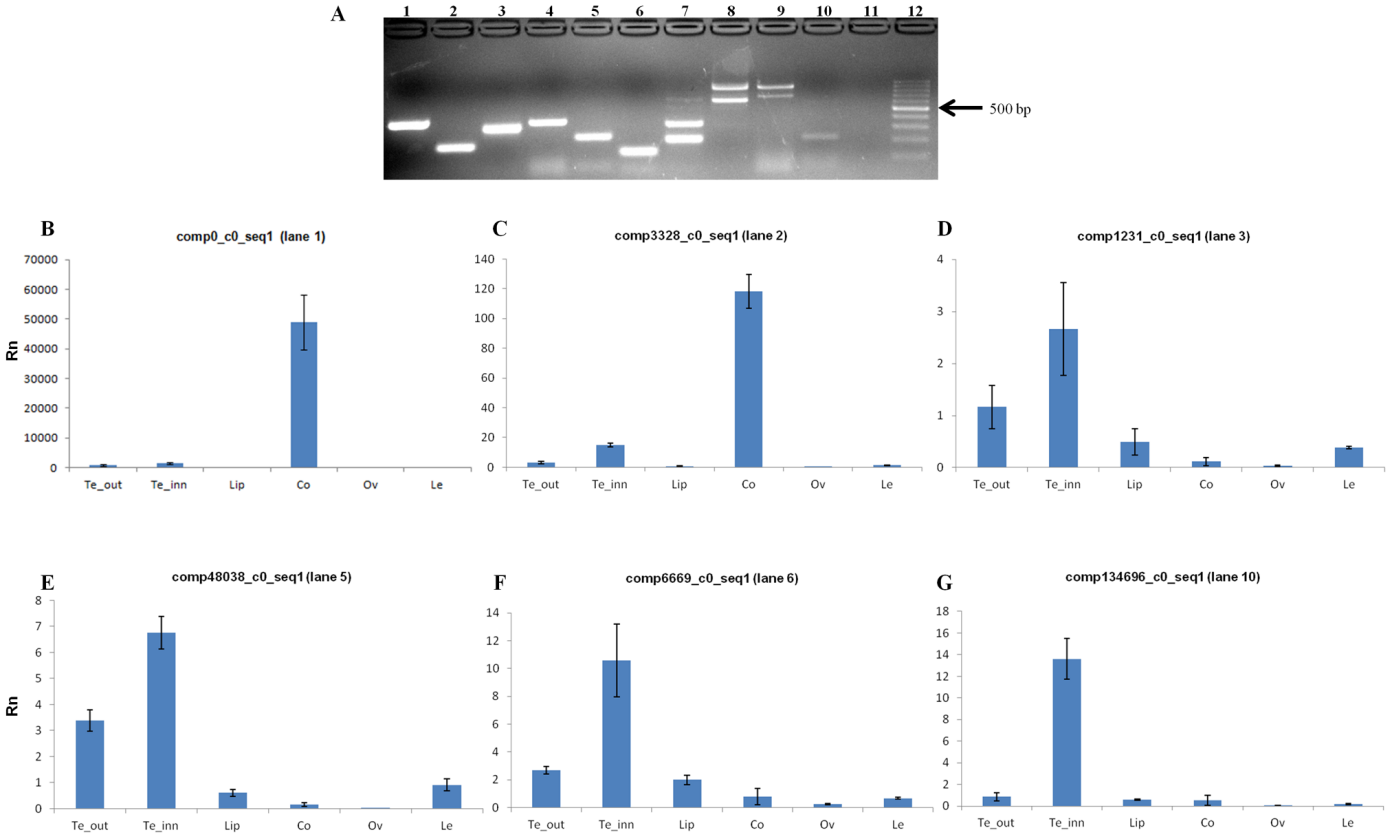
Selected putative long non-coding RNAs expressed in the inflorescence of *O. italica*. (**A**) Agarose gel electrophoresis of the RT-PCR-amplified products of the selected transcripts. Lane 1, comp0_c0_seq1; lane 2, comp3328_c0_seq1; lane 3, comp1231_c0_seq1; lane 4, comp3311_c0_seq1; lane 5, comp48038_c0_seq1; lane 6, comp6669_c0_seq1; lane 7, comp4129_c0_seq1; lane 8, comp1308_c0_seq1; lane 9, comp15481_c0_seq1; lane 10, comp134696_c0_seq1; lane 11, empty; lane 12, 100 bp ladder. (**B–G**) Relative expression level (Rn) in the outer tepals (Te_out), inner tepals (Te_inn), labellum (Lip), column (Co), ovary (Ov) and leaf (Le) of the transcripts comp0_c0_seq1 (**B**), comp3328_c0_seq1 (**C**), comp1231_c0_seq1 (**D**), comp48038_c0_seq1 (**E**), comp6669_c0_seq1, (**F**), and comp134696_c0_seq1 (**G**). The bars indicate the standard deviation.

**Figure 9:**
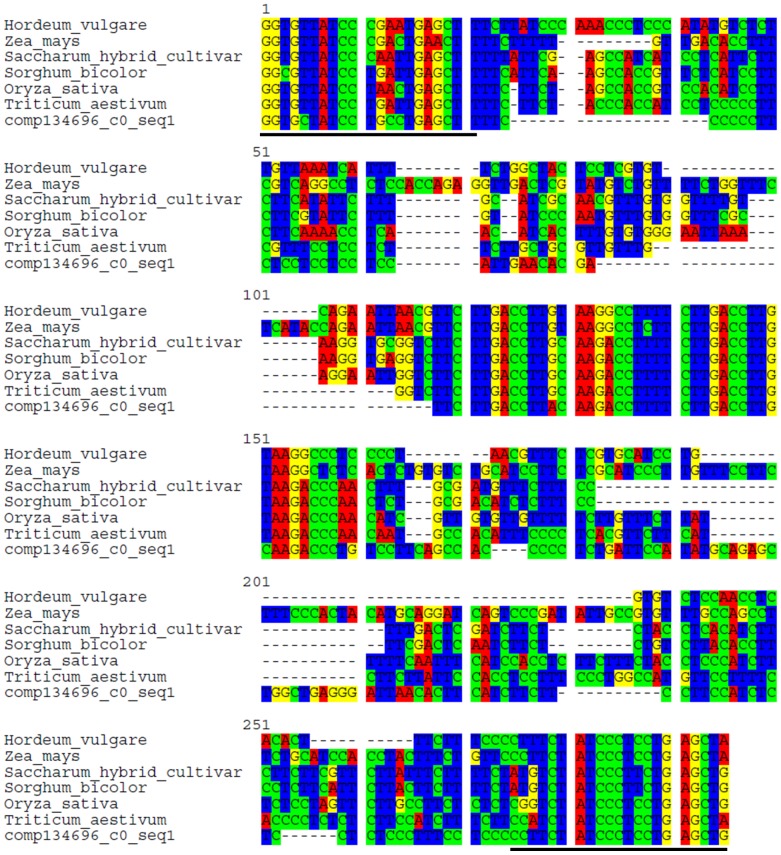
Nucleotide sequence alignment of comp134696_c0_seq1 of *O. italica* and the *TAS3* sequences of *Hordeum vulgare* (accession number BF264964), *Zea mays* (BE519095), *Saccharum hybrid cultivar* (CA145655), *Sorghum bicolor* (CD464142), *Oryza sativa* (AU100890), and *Triticum aestivum* (CN010916). The 5′ and 3′ conserved sequences that are targets of miR-390 are underlined.

RT-PCR amplification was conducted on total RNA extracted from inflorescence of *O. italica*, resulting in an amplification product of the expected size for 7 of the 10 analyzed transcripts (Figure 8 A). Multiple fragments were obtained for 3 transcripts (Figure 8 A, lane 7–9) including the 2 long transcripts that matched only the CPC threshold. The 7 amplification products of the expected size were cloned and sequenced; six of the sequences were successfully confirmed, while 1 resulted from a contaminant sequence (Figure 8 A, lane 4). Real-time PCR experiments were performed to analyze the expression pattern of these 6 non-coding transcripts in different floral tissues and leaf of *O. italica* (Figure 8 B–G). All the transcripts were differentially expressed in the examined tissues, absent in the ovary and absent (comp0_c0_seq1, comp3328_c0_seq1, comp134696_c0_seq1) or weakly expressed (comp1231_c0_seq1, comp48038_c0_seq1, comp6669_c0_seq1) in the leaf. The comp0_c0_seq1 and comp3328_c0_seq1 transcripts (Figure 8 B and C, respectively) were mainly expressed in the column, suggesting a possible role in male and female reproductive tissues. The comp1231_c0_seq1, comp48038_c0_seq1 and comp6669_c0_seq1 transcripts (Figure 8 D–F, respectively) exhibited lower expression than comp0_c0_seq1 and comp3328_c0_seq1 and were restricted almost exclusively to the tepals (outer, inner and lip). The comp134696_c0_seq1 transcript (Figure 8 G) was expressed in inner tepals and seemed to be almost absent in the other tissues. The presence of specific putative lncRNAs in the tepals of *O. italica* suggests they could have a functional role in these organs. In addition, a BLASTN search revealed that comp134696_c0_seq1 is a homolog of the *TAS3* long non-coding transcript (Figure 9). *TAS3* is the precursor transcript of several *trans*-acting small interfering RNAs (ta-siRNAs), a plant-specific family of small RNAs [70]. In many plant species, the *TAS3* transcript is targeted and cleaved in two conserved positions by the microRNA phase-initiator miR-390. The resulting transcript is converted into double-strand RNA by the RNA-dependent RNA polymerase RDR6 and subsequently cleaved into ∼21 nt sRNAs by the Dicer-like enzyme DCL4. The ta-siRNAs produced by the *TAS3* locus bind the ARGONAUTE (AGO) proteins and direct the cleavage of transcripts of the auxin response factor genes [70], [71]. In the inflorescence transcriptome of *O. italica* there are functionally annotated transcripts that correspond to RDR6 (comp44794_c0_seq1), DCL4 (comp3192_c0_seq1) and 11 transcripts matching different AGO proteins. In addition, the homolog of miR-390 is differentially expressed in the various tissues of the inflorescence of *O. italica* [17]. These results suggest the existence of a conserved pathway for the *TAS3* ta-siRNA biogenesis in the inflorescence of *O. italica*. The question arises of whether the *TAS3* ta-siRNAs are actually present in the inflorescence of *O. italica* and whether they have a role in this tissue, since in other plant species they are involved in lateral roots development, leaf morphology, juvenile-to-adult stage transitions and the response to pathogens [72], [73], [74].

## Conclusions

The assembled transcriptome of *O. italica* increases the RNA-seq data currently available for orchids, specifically for the Orchidoideae sub-family. The NGS approach was employed for the first time in orchids to identify putative lncRNAs expressed in the floral organs, opening a challenging field of investigation in these non-model plant species. Previous studies revealed the regulatory function of a small non-coding RNA (miR-5179) on *OitaDEF2*, a *DEF*-like gene of *O. italica* [17]. In orchids, the MADS-box *DEF*-like genes are involved in the diversification of the orchid perianth, as explained by the “orchid code” theory [75], [76]. Our results indicate that also some long non-coding transcripts are flower-specific and differentially expressed in the different tissues of the perianth of *O. italica* and, if confirmed also in other orchid species, suggest they might be relevant for flower development.These evidences strongly encourage to focus the transcriptomic and genomic studies towards lncRNAs, both in model and non-model plant species, to clarify their possible role in the different biological processes.

## Supporting Information

Table S1 Annotation table of the assembled unigenes of *O. italica*.
(XLSX)

Table S2 GO annotation of the assembled unigenes of *O. italica*.
(XLSX)

Table S3 Pfam domain annotation of the assembled unigenes of *O. italica*.
(XLSX)

Table S4 FPKM counts of the assembled unigenes of *O. italica*.
(XLSX)

Table S5 GO annotation of the 1,144 most expressed unigenes of *O. italica* and comparison of their level 2 GO terms with those of the reference transcriptome.
(XLSX)

Table S6 Long non-coding RNA predictions.
(XLSX)

File S1 Assembled unigenes of *O. italica*.
(7Z) Click here for additional data file.
